# A Rare Case of Primary Posterior Abdominal Wall Muscle Hydatid Cyst With Thoracolumbar Spinal Extension

**DOI:** 10.7759/cureus.61198

**Published:** 2024-05-27

**Authors:** Rajshree U Dhadve, Vishav Bir S Thakur, Suhas M, Divyajat Kumar

**Affiliations:** 1 Department of Radiodiagnosis, Dr. D. Y. Patil Medical College, Hospital and Research Centre, Dr. D. Y. Patil Vidyapeeth (Deemed to be University), Pune, IND

**Keywords:** spinal extension, hydatid cyst, neural foramina, muscular, posterior abdomen wall

## Abstract

Primary intramuscular hydatid cysts are uncommon due to the contractile nature of muscles and their lactic acid content. Hydatid cysts with spinal extension are sometimes seen with primary vertebral body involvement. Our patient presented with a slow-growing posterior abdominal wall mass, and upon magnetic resonance imaging (MRI), it was revealed to be several cystic lesions in the abdomen wall with extension through the neural foramina into the spinal canal. The key differentials for spinal canal masses with neural foraminal expansion and muscle involvement are peripheral nerve sheath tumors. Our case report adds hydatid cysts to the differentials for well-defined cysts with variable intensities on MRI.

## Introduction

Echinococcosis, or hydatid cyst disease, is a zoonotic parasitic infection that is transmitted from animals to humans. The condition is attributed to the larvae of cestodes belonging to the genus Echinococcus and the family Taeniidae [1]. Carnivores serve as the primary hosts and shelter the fully developed tapeworm. Herbivores, on the other hand, act as intermediate hosts by consuming the eggs, from which the embryos are released. Humans, however, unintentionally, also function as intermediate hosts [2]. The tapeworm cyst can be located in any part of the body; however, it is most frequently detected in the liver (55%-70%) and lungs (20%-30%). The disease seldom affects the internal organs, such as the heart, kidneys, spleen, and brain. The skeletal muscles are affected even less commonly, occurring in about 1% to 4% of patients [3]. Very few cases of hydatid cysts involving the extension of paraspinal hydatid cysts into the spinal canal through the neural foramen without causing any damage to the surrounding bone have been documented in the literature [4,5]. Based on our understanding, encountering a primary hydatid cyst with multiple daughter cysts in the posterior abdominal wall and paraspinal muscles is quite uncommon. Furthermore, one of the daughter cysts extends beyond the vertebral foramen, causing compression of the spinal cord, adding to its rarity.

## Case presentation

A 57-year-old male patient, a resident of a rural central region of Maharashtra, was admitted to the neurosurgical ward with chief complaints of paresthesia of bilateral lower limbs for two months, with the left lower limb being predominantly affected. The patient gave a history of swelling in the right thoracolumbar region for seven years, with a gradual increase in size, and complained of diffuse pain in the last three years. Physical examination revealed a swelling of size 40 cm x 15 cm along the right midback in the thoracolumbar paravertebral region. The swelling was immobile and hard in consistency, without any local rise in temperature or tenderness. The skin overlying the swelling was normal. On neurological examination, he was conscious and well-oriented; both lower limbs had a power of 3/5 with brisk knee and ankle tendon reflexes bilaterally, without sphincteric disorders, a negative Babinski sign, or any other sensory involvement. The rest of the physical examination was unremarkable. There is a past history of contact with domesticated animals dating back 15 years, which included the rearing of goats in his household and contact with stray canines in his native village. No significant surgical, medical, or family history was present. Laboratory tests, including hemoglobin, renal function tests, and liver function tests, were within the normal range.

The patient initially underwent ultrasonography of the abdomen and pelvis, which revealed a large, ill-defined, heterogeneously hyperechoic, solid-cystic, predominantly cystic lesion located in the retroperitoneal region, specifically in the right paravertebral space, adjacent to the right psoas muscle, displacing it anteromedially. The solid component of the lesion showed vascularity on Doppler. A contrast-enhanced computed tomography scan (CECT) of the thorax, abdomen, and pelvis was performed for further evaluation.

**Figure 1:**
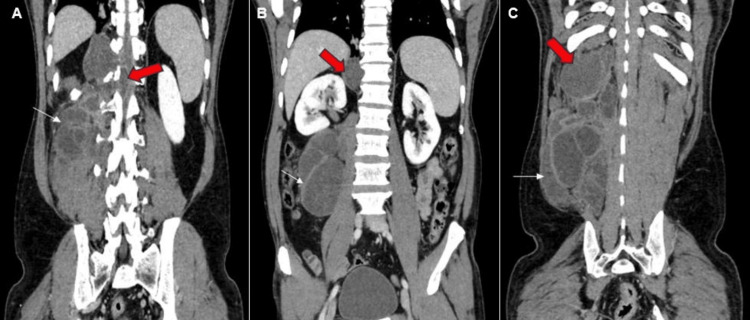
Reconstructed coronal sections of contrast enhanced computed tomography scan of the abdomen and the pelvis. (A) Intraspinal extension of the hydatid cyst (red block arrow). Multiple cysts are seen in the paraspinal muscles (white arrow). (B) Superior extension of the cysts displacing the right kidney (red block arrow). Posterior abdomen wall and paraspinal muscle showing multiple cysts (white arrow). (C) Superior extent of the lesion reaching the caudal surface of the diaphragm (red arrowhead) and multiple daughter cysts (white arrow).

**Figure 2:**
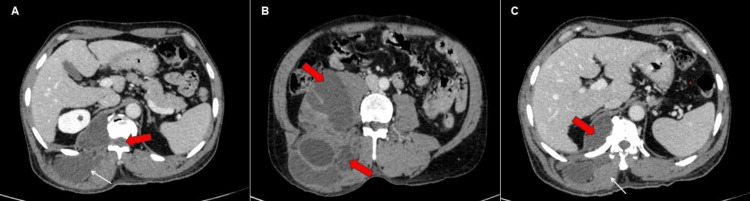
Contrast-enhanced computed tomography of the abdomen and pelvis in axial sections. (A) Intraspinal extension of the hydatid cyst (red block arrow). Multiple cysts are seen in the paraspinal muscles (white arrow). (B) Posterior abdomen wall and paraspinal muscle showing multiple cysts (red block arrows). (C) Daughter cysts causing mass effect in the form of mild scalloping of the adjacent vertebral body (red arrowhead) and medial displacement of the erector spinae muscle (white arrow).

On a CECT scan of the abdomen and pelvis, a large, fairly well-defined, multi-loculated, predominantly cystic lesion is revealed, primarily involving the right posterior abdominal wall and paraspinal muscles, with septations showing post-contrast enhancement, as depicted in Figures 1-2. Additionally, it extends medially through the neural foramina.

On the basis of CECT findings, plexiform neurofibroma and hydatid cysts were suspected, and MRI was suggested for further evaluation. 

**Figure 3:**
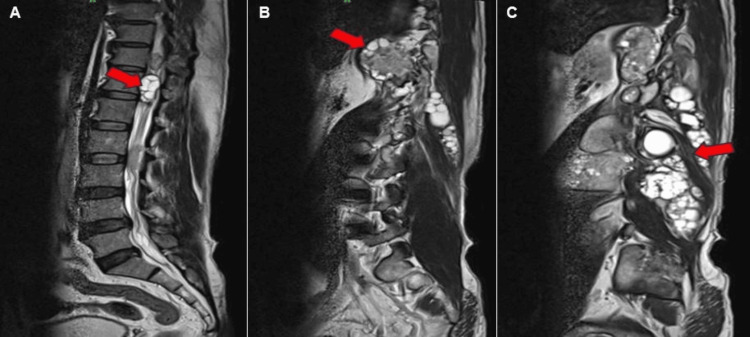
Magnetic resonance imaging of T2-weighted sagittal and parasagittal images of the thoracic and lumbosacral regions. (A) Intraspinal extension of the cysts (red block arrow). (B) Paraspinal muscle involvement with a pleuroperitoneal excursion along with multiple daughter cysts (red block arrow). (C) Multiple daughter cysts (red block arrow) are seen within the mother cyst in right quadratus lumborum and erector spinae muscles.

On MRI of the lumbosacral spine, the lesion exhibited typical characteristics of a hydatid cyst, including the presence of several daughter cysts within the mother cyst. In Figure 3, the daughter cysts exhibited low signal intensity on T1-weighted images and high signal intensity on T2-weighted images.

**Figure 4:**
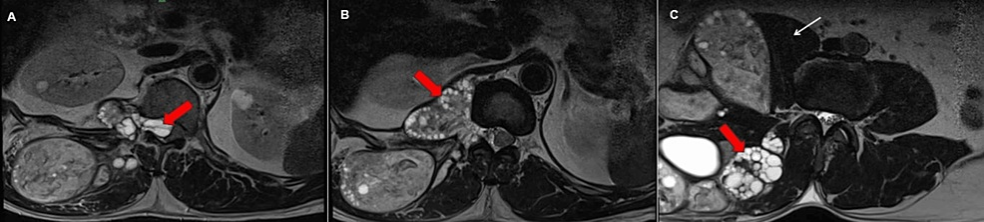
Magnetic resonance imaging of T2-weighted axial images of the abdomen and pelvis. (A) Intraspinal extension of the cysts (red block arrow). (B) Paraspinal muscle involvement with a pleuroperitoneal excursion along with multiple daughter cysts (red block arrow), causing mild scalloping of the adjacent vertebral body. (C) Multiple daughter cysts (red block arrow) are seen within the mother cyst in right quadratus lumborum and erector spinae muscles. Right psoas muscle (white arrow) is displaced anteromedially.

Figure 3 shows the sagittal and parasagittal sections. Figure 4 shows axial sections of the abdomen and pelvis Figures 3-4 showcase T2-weighted MRI scans, showing daughter cysts with spinal extension. 

MRI of the cysts shows extension through the neural foramina and cord compression, prompting the suggestion of a biopsy for a definitive diagnosis.

**Figure 5:**
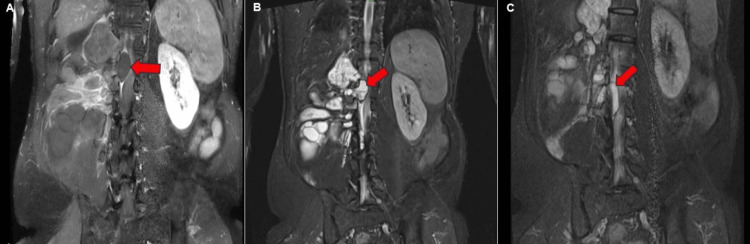
Magnetic resonance imaging of T1-weighted images with post-contrast enhancement and short tau inversion recovery images in the coronal section. (A) Post-contrast T1-weighted magnetic resonance imaging shows thin peripheral enhancement of the cysts (red block arrow). (A-C) Intraspinal extension causing the left lateral displacement and compression of the spinal cord (red block arrow). (B, C) Short tau inversion recovery magnetic resonance images showing cysts extending through the neural foramina, with intraspinal extension causing cord compression (red block arrow).

Figure 5 shows T1-weighted post-contrast and short tau inversion recovery MRI scans showing extension of the daughter cysts. 

**Figure 6:**
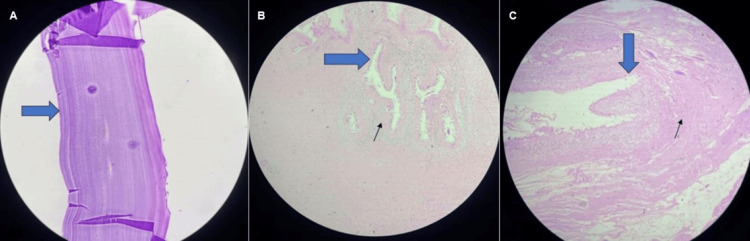
Histopathological examination of the biopsy: hematoxylin and eosin-stained images. (A) Original magnification x40 - a photomicrograph shows the acellular part of the lamellated membrane of the hydatid cyst. (B) The scanogram shows a cyst wall composed of an acellular laminated membrane (blue solid arrow), along with areas of granulation tissue and chronic inflammatory infiltrates (black arrow). (C) Original magnification x20 shows Protoscolex (blue block arrow) invading the muscle layers (black arrow).

Intraoperatively, multiple cysts were visualized, and a biopsy was taken for histopathological examination (HPE). On HPE, the diagnosis was confirmed to be a hydatid cyst. The cyst wall exhibited a lamellated appearance, along with chronic granulation tissue and scolex attachment to the muscle fibers, as depicted in Figure 6.

## Discussion

Humans serve as unintentional intermediate hosts in the life cycle of the Echinococcus tapeworm. The most important source of the infection is the livestock reared in grass fields around the world. The larvae of the tapeworms Echinococcus granulosus, Echinococcus multilocularis, and Echinococcus vogeli are the most prevalent species that infect humans [6,7]. Following ingestion by humans, the protective shell of the E. granulosus ova gets eliminated, and it develops into an oncosphere larvae, which successfully infiltrates the duodenal mucosa. It then proceeds to enter the portal circulation, spreading to the liver and subsequently to the lungs. After three weeks, the embryo undergoes development and transforms into a larva while producing a cyst [8].

The liver is the most common location for hydatid cysts, accounting for 75% of cases. This is due to the passage of blood from the intestine through the portal circulation. The lungs are the second most common site, representing 10% of cases. Less than 3% of cases are attributed to intramuscular hydatid cysts [9]. Due to the contractile properties of muscles and their high lactic acid content, they are not a viable location for the implantation of hydatid cysts [9]. Even among different muscle groups, paraspinal muscles are less commonly impacted [10,11]. There have been a few recorded occurrences of paraspinal muscle hydatid cysts extending into the vertebral foramen. The etiology of hydatid cysts in muscles remains uncertain. Several hypotheses have been put forth. Certain studies claim that the larva may come into direct contact with the wound, such as in the case of a dog bite, which could potentially explain the phenomenon. Conversely, others suggest that the larva is transmitted to the muscles through the bloodstream from the primary source in the liver and lungs, accounting for approximately 10% to 15% of instances [12]. Alternatively, some individuals believe that larvae are transferred through the muscular layer of the gut and into the venous system without going through the liver [13,14].

The clinical manifestations of a hydatid cyst range from the existence of a swelling or mass to symptoms caused by pressure, cyst infection, and, in rare cases, anaphylactic shock [15]. The hydatid cyst of muscle usually presents as a painless, gradually expanding, and asymptomatic mass with intact skin. Therefore, it is crucial to maintain a high level of suspicion, as the potential causes of this condition might vary from chronic hematoma and lipoma to malignant soft tissue tumors like myxoid liposarcoma [10].

The biological tests indicate the presence of hyper-eosinophilia, although they are inconsistent as they are found in only 25% of patients and lack specificity; therefore, positive serologic testing is useful [16]. However, in 50% of instances of primary intramuscular hydatidosis, serology yields false-negative results due to the capsule's ability to shield the parasite from the host's immune system [15].

In our case, the patient exhibited chronic, slowly advancing swelling in the right posterior lumbar region. He showed recent neurological symptoms of bilateral tingling sensations, together with slightly decreased motor function in the right lower limb. Radiological imaging findings revealed a multiloculated cystic lesion with daughter cysts within, raising suspicion of a hydatid cyst. MRI is the investigation of choice for identifying hydatid cysts. It provides a clear visualization of a cyst with a thin wall and multiple daughter cysts within the mother cyst [11].

Ultrasonography is the preferred imaging technique for identifying muscle hydatid cysts due to its noninvasive nature, although MRI is now regarded as the investigation of choice due to its improved contrast resolution and multiplanar slices [17]. Muscular hydatid cysts can be identified on MRI images by a clear cystic mass with a thin cystic wall and membrane that are separated from the mass [17].

The primary treatment for a solitary parasitic cyst or multiple cysts found in the subcutaneous or muscle tissue is complete surgical removal. This procedure is necessary to avoid serious consequences resulting from the presence of progressing or ruptured cysts [18]. Antihelminthic medications, such as albendazole, are regularly given both before and after the surgical procedure [19]. The risks associated with leakage, such as allergic responses and recurrence following surgery, must be acknowledged [18]. The most effective treatment for musculoskeletal hydatid disease involves surgical removal in conjunction with anthelmintic chemotherapy; the latter reduces the number of live cysts and the risk of recurrence [13].

## Conclusions

To the best of our knowledge, this is a rare instance of echinococcal cysts being extensively distributed throughout the quadratus lumborum and erector spinae muscles. The cysts traversed the thoracolumbar vertebral foramen, causing widening of the neural foramina with scalloping of the vertebral body and exerting pressure on the spinal cord, resulting in sensory and motor complaints. In summary, hydatid cysts infrequently occur in muscles such as the quadratus lumborum and erector spinae, along with extension within the spinal foramen. The primary diagnostic methods include imaging by radiology and biopsy procedures. In such circumstances, we consider surgery combined with ongoing medical therapy of albendazole to be the most effective option for preventing recurrence. As the epicenter of the lesion was primarily within the muscle with a small neural foraminal component, we labeled it as a primary intramuscular hydatid with spinal extension.
